# Massive Subcutaneous Emphysema, Pneumoperitoneum, Pneumoretroperitoneum, and Pneumoscrotum following Endoscopic Retrograde Cholangiopancreatography in a Living Liver Donor

**Published:** 2018-08-01

**Authors:** S. Akbulut, B. Isik, Y. Karipkiz, S. Yilmaz

**Affiliations:** 1 Department of Surgery and Liver Transplant Institute, Inonu University Faculty of Medicine, 244280, Malatya, Turkey; 2 Department of Nursing Service, Inonu University Faculty of Medicine, 244280, Malatya, Turkey

**Keywords:** Living donor hepatectomy, Biliary complication, ERCP-related complication, Duodenal perforation

## Abstract

Despite having many advantages, living donor liver transplantation has not been adopted by western countries due to risk of nearly life-threatening complications after living donor hepatectomy (LDH). Herein, we aimed at presenting the management of a 19-year-old patient who suffered life-threatening complications after right lobe LDH. A multiple detector computed tomography (MDCT) revealed a bilioma at the cut surface of the remnant liver, for which a transhepatic drainage catheter was placed. Endoscopic retrograde cholangiopancreatography (ERCP) performed to decompress biliary tract, but the biliary tract could not be cannulized due to post-precut bleeding. On the next day, extensive crepitation was detected and MDCT showed subcutaneous emphysema, pneumoperitoneum, pneumoretroperitoneum, and pneumoscrotum (ERCP-related duodenal perforation?). However, the patient showed significant deterioration of physical examination findings, fever, and infectious parameters, and therefore was taken to the operating room. Kocher maneuver revealed no apparent duodenal perforation. Then, a 2-mm bile duct was found open at the caudate lobe, through which bile leaked. Then, common bile duct exploration and T-tube placement were performed, followed by suture closure of the bile orifice at the caudate lobe. Massive air previously identified completely disappeared one week after the operation.

## INTRODUCTION

Since the first successful liver transplantation by Starzl and colleagues in 1967, liver transplantation has become a standard therapy for a variety of liver disorders including chronic liver disease, acute liver failure, metabolic liver disorders, and some liver tumors [1]. While deceased donor liver transplantation constitutes a large percentage of liver transplantation performed in western countries, the majority of liver transplantation performed in many of the Asian countries use organs procured from living donors mainly due to donor pool shortage [1, 2]. Despite having many advantages, living donor liver transplantation (LDLT) has not been adopted by western countries due to complications of living donor hepatectomy (LDH), of which some may be fatal [3-5]. Therefore, biliary tract complications are the leading ones among most fearful complications by surgeons. Herein, we aimed at sharing the management of a healthy donor candidate’s misfortunes experienced due to biliary tract complications of LDH.

## CASE PRESENTATION

**Figure 1:**
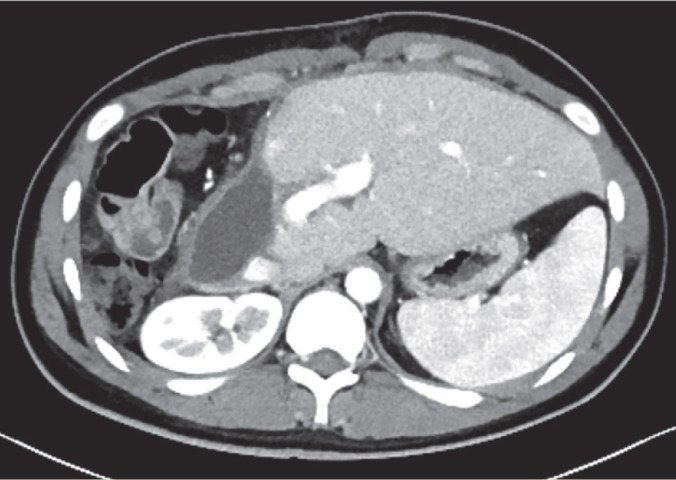
The axial CT image shows a collection consistent with a bilioma on the cross-sectional surface of the remnant liver

**Figure 2:**
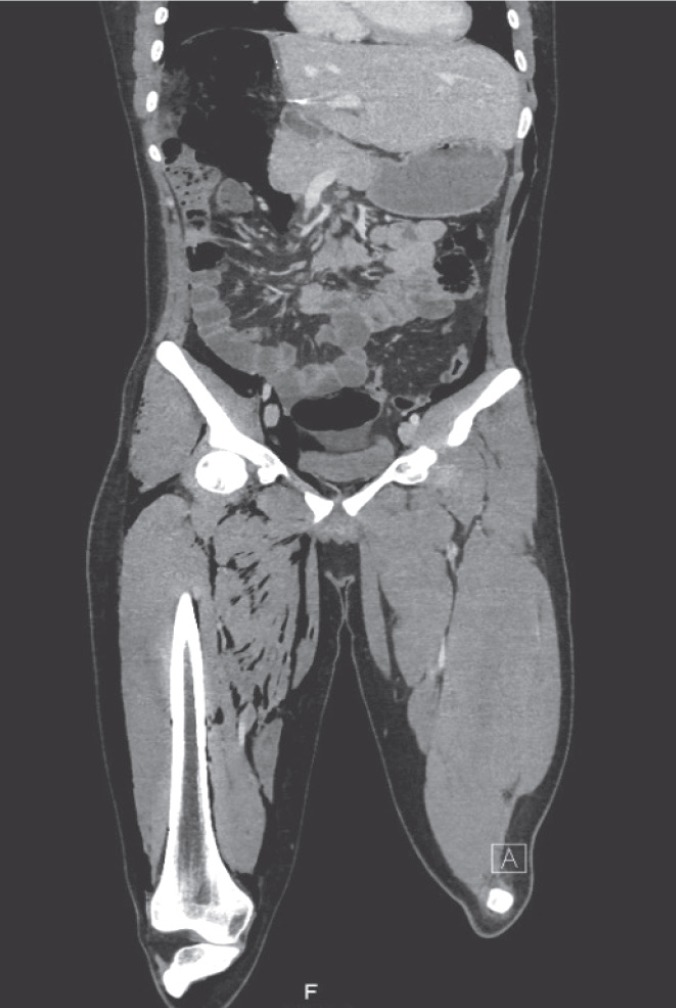
The coronal CT cross-section demonstrates massive subcutaneous emphysema, pneumoperitoneum and pneumoretroperitoneum

**Figure 3:**
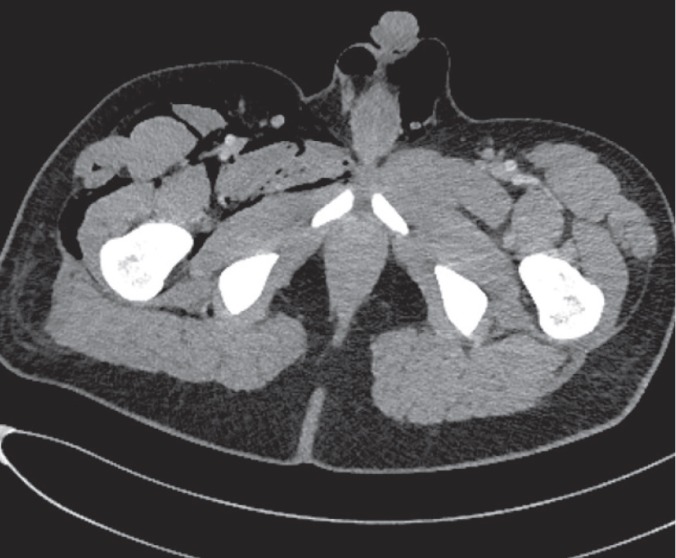
The axial CT image shows pneumoscrotum

**Figure 4:**
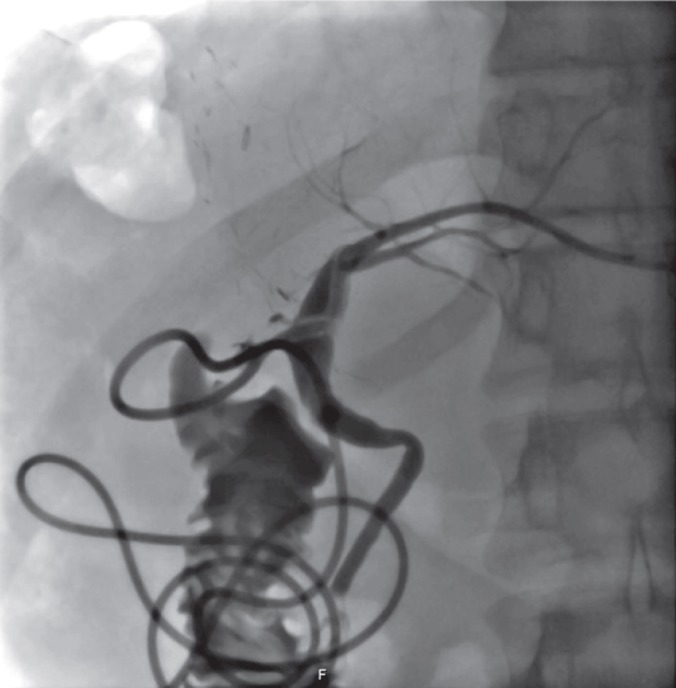
A cholangiogram taken on the 10th post-operative day showing a non-dilated biliary tract and the absence of any biliary leak

A 19-year-old donor candidate (remnant volume: 30%, BMI: 26.2 kg/m^2^, O^+^) presented to our center to donate liver to her grandmother who had chronic liver failure secondary to hepatitis B. The patient underwent standard right lobe LDH. An abdominal contrast-enhanced computed tomography (CT) performed for post-operative fever and leukocytosis revealed a collection consistent with a bilioma in a location close to the liver cut surface (Fig 1). The patent was first put on imipenem treatment and then placed a percutaneous transhepatic bilioma drainage catheter-guided percutaneous transhepatic cholangiography (PTC). An Endoscopic retrograde cholangiopancreatography (ERCP) was performed both to decompress the biliary tract and to control biliary leak, but the procedure failed due to bleeding during sphincterotomy. One day after ERCP crepitations were noticed in the patient’s scrotum, abdominal wall, and right thigh. The signs were interpreted as a result of an iatrogenic duodenal perforation due to ERCP. A contrast-enhanced CT showed subcutaneous emphysema, pneumoperitoneum, pneumoretroperitoneum, and pneumoscrotum (Figs 2, 3). Therefore, a treatment protocol consisting of nasogastric decompression, fluid and electrolyte replacement, and wide spectrum antibiotics (imipenem, fluconazole, daptomycin) was started. On the third day of the treatment, the patient developed fever (38.5 °C), rise in CRP (18.9 mg/dL), leukocytosis (17,600/L) and hematemesis, and was taken to the operating room. The abdominal cavity was entered through the old incision. The Kocher maneuver was performed to check the second and third parts of the duodenum, which revealed no apparent perforation. During hilus dissection, a biliary leak was realized from a bile duct with a diameter of approximately 2 mm in the remnant caudate lobe. A cholangiogram taken by administering contrast material through the cystic canal revealed normal remnant biliary tree. A cholangiogram taken by administering contrast material through the open biliary duct in the caudate lobe showed that the contrast material passed to a small part of the caudate lobe. The bile duct in the caudate lobe was sutured. In order to decompress biliary tract effectively, common bile duct exploration with T-tube insertion were carried out. Clinical signs and symptoms dramatically improved at the post-operative period. Upon showing the absence of biliary leak in a cholangiogram taken on the 10^th^ day, the T-tube was put under a pad and the patient was discharged (Fig 4). The T-tube was removed as no leak was observed at six-week control.

## DISCUSSION

Post-LDH complications can be categorized into biliary and non-biliary complications. The overall and biliary complication rates after LDH are 8.1%–50%, and 1.9%–18%, respectively [3]. There are simple wound infection and atelectasis on the one end of the spectrum of non-biliary complications and life-threatening complications such as post-operative bleeding, thromboembolism, portal vein thrombosis, severe pancreatitis, and gastrointestinal perforation on the other end [4]. Bilioma that improves spontaneously without intervention is on one end of the biliary complication scale, and biliary leaks that persist despite all sorts of invasive procedures on the other end. In a study by Braun, et. al. [3], which evaluated the outcomes of 12,653 donors suggested that biliary leaks and biliomas that cause no deterioration of clinical parameters may be followed conservatively without performing any invasive treatment such as (ERCP, PTC). On the other hand, biliary strictures that are more rarely encountered mostly require ERCP and stenting [4]. It is an established fact that the ERCP used for complication management also causes its own complications. Although there exist several studies reporting complications like pancreatitis, bleeding, and perforation secondary to post-liver transplantation ERCP, no ERCP-related complication has ever been reported to occur in living liver donors [6]. To the best of our knowledge, this is the first case of ERCP-related perforation in the literature. 

In conclusion, this case report demonstrates how completely healthy living hepatic donors may suffer life-threatening complications. The timing of complication management is as important as its mode in such cases.
